# Supplementary material for: Content-rich biological network constructed by mining PubMed abstracts
Source: BMC Bioinformatics. 2004 Oct 8;5:147. doi: 10.1186/1471-2105-5-147 (PMC528731; doi:10.1186/1471-2105-5-147)
Supplement: Additional File 2 — The original results of the above study (non-essential files are deleted to keep the file size under the limit set by BMC bioinformatics). [file 1471-2105-5-147-S2.bz2 › chilibotAdditionalFile2/dip40/397ID8663535E9951/html/EGFR_AREG.html]

 


 **EGFR** and **AREG** 
  
Found 112 abstracts in PubMed, retrieved 40.  
 

 What does Google say? 
 PDF only 
| .edu only 

---

**Interactive relationship** (e.g. stimulation, inhibition, etc)

**Neutral relationship**- We found that amphiregulin  [ **AREG** ]  AR was the most abundant  **EGFR**  ligand expressed in HCA 7 and Caco 2 cells.  Ref: 10362109 Br J Cancer, 1999
- Treatment with either TGFalpha or amphiregulin  [ **AREG** ]  resulted in higher c erbB1  [ **EGFR** ]  activation and c erbB3 levels.  Ref: 12202942 Urol Res, 2002
- These cells express both transforming growth factor alpha and amphiregulin  [ **AREG** ]  and require autocrine signaling through the  **EGFR**  for proliferation and migration.  Ref: 10339571 Proc Natl Acad Sci U S A, 1999
- ... the activation of  **EGFR**  in response to smoke involves cleavage of amphiregulin  [ **AREG** ]  by ADAM 17.  Ref: 12568494 Novartis Found Symp, 2002
- Expression of amphiregulin  [ **AREG** ]  and epidermal growth factor receptor  [ **EGFR** ]  in human breast cancer analysis of autocriny and stromal epithelial interactions.  Ref: 11523048 J Pathol, 2001
- In support of this, transforming growth factor alpha, EGF and amphiregulin  [ **AREG** ]  mRNAs were detected by RT PCR in the epidermal growth factor receptor  [ **EGFR** ]  expressing cell lines.  Ref: 11875715 Br J Cancer, 2002
- The epidermal growth factor receptor  [ **EGFR** ]   **EGFR**  mediates the actions of a family of bioactive peptides that include epidermal growth factor EGF and amphiregulin  [ **AREG** ]  AR.  Ref: 10085134 J Biol Chem, 1999
- AG1478 decreased the expression of genes for amphiregulin  [ **AREG** ] , HB EGF and betacellulin, showing that  **EGFR**  activation induces up regulation of the  **EGFR**  ligands.  Ref: 11133810 Carcinogenesis, 2000
- Amphiregulin  [ **AREG** ]  increased expression of c erbB1  [ **EGFR** ] .  Ref: 12202942 Urol Res, 2002
- RESULTS Oxidative stress and osmotic stress induced tyrosine phosphorylation of  **EGFR**  within 2 minutes, followed by a marked increase in HB EGF and amphiregulin  [ **AREG** ]  transcripts in RGM1 cells.  Ref: 11208719 Gastroenterology, 2001

**Non-interactive relationship** (e.g. studied together, co-existance, homology, etc.)

- Consistent with these profiles, recent gene knockouts established that  **EGFR**  and its ligand, Amphiregulin  [ **AREG** ]  AR, are essential for ductal morphogenesis in the adolescent mouse and likely provide the required epithelial stromal signal.  Ref: 11467454 J Mammary Gland Biol Neoplasia, 2001
- In this study, we compared expression levels of messenger RNAs mRNAs encoding the epidermal growth factor EGF receptor related receptor tyrosine kinases ErbB1  [ **EGFR** ]  through 4, the six EGF receptor ligands, EGF, transforming growth factor TGF alpha, amphiregulin  [ **AREG** ]  ARG, HB EGF, betacellulin, and epiregulin, and the related molecule heregulin alpha, in a series of 10 prostate tissue specimens.  Ref: 10579352 Endocrinology, 1999
- There were no significant associations with cytoplasmic or membrane immunoreactivity, but cases showing nuclear expression in >25% of cells were associated with good histological grade, epidermal growth factor receptor  [ **EGFR** ]  expression, c erbB  [ **EGFR** ]  3 positivity, cripto, amphiregulin  [ **AREG** ] , and transforming growth factor alpha overexpression.  Ref: 10749108 Cancer Res, 2000
- The binding of amphiregulin  [ **AREG** ]  to  **EGFR**  then stimulates proliferation of lung epithelial cells.  Ref: 12711607 J Biol Chem< MedlineTA>J Biol Chem, 2000
- The expression of three other ligands for  **EGFR**  epidermal growth factor, cripto, and amphiregulin  [ **AREG** ]  was examined by Northern analysis to determine whether they might also contribute to a potential growth stimulatory loop.  Ref: 9815714 Clin Cancer Res, 1997
- Amphiregulin  [ **AREG** ]  AR and its receptor, epidermal growth factor receptor  [ **EGFR** ]   **EGFR** , were evaluated by dual immunostaining in a series of 84 invasive ductal breast carcinoma specimens, 33 of which were from locally advanced inflammatory T4d cancer.  Ref: 11523048 J Pathol, 2001
- Similarly,  **EGFR**  TK inhibition by ZD1839 reversed the invasive phenotype promoted by the constitutively activated form of the  **EGFR**  EGFRvIII and the  **EGFR**  agonists transforming growth factor alpha TGFalpha, amphiregulin  [ **AREG** ]  and EGF.  Ref: 12881705 Oncogene, 2003
- We demonstrate that mRNAs for the 6 major c erbB  [ **EGFR** ]  ligands, namely, epidermal growth factor EGF, transforming growth factor alpha TGF alpha, betacellulin BTC, heparin binding epidermal growth factor like growth factor HB EGF, amphiregulin  [ **AREG** ]  AR and heregulin HRG, are expressed in a large panel of HNSCC cell lines.  Ref: 11072245 Int J Cancer, 2000
- This cell line was shown to have a higher expression of EGF receptors  **EGFR**  and amphiregulin  [ **AREG** ]  AR when compared to their normal counterparts or less aggressive transformed cells.  Ref: 10597254 Oncogene, 1999
- The smoke triggered mechanism includes the generation of oxygen radicals, which in turn stimulate tumor necrosis factor alpha converting enzyme TACE, ADAM 17 metalloproteinase to cleave transmembrane amphiregulin  [ **AREG** ] , a ligand for the epidermal growth factor receptor  [ **EGFR** ]   **EGFR** .  Ref: 12711607 J Biol Chem< MedlineTA>J Biol Chem, 1999
- Little is known about the regulative role of the four other widely expressed epidermal growth factor receptor  [ **EGFR** ]  ligands, heparin binding EGF, amphiregulin  [ **AREG** ] , betacellulin and cripto in the gastric mucosa.  Ref: 11218236 Scand J Gastroenterol, 2001
- Our results point to the involvement of several ErbB  [ **EGFR** ]  specific ligands amphiregulin  [ **AREG** ]  and neuregulin 1 and enzymes or adaptor molecules PI3K, Src, Shc and Grb7 in the ErbB  [ **EGFR** ]  pathway dysregulation associated with breast cancer.  Ref: 12866037 Int J Cancer, 2003
- RT PCR transcripts of  **EGFR**  ligands EGF, TGF alpha, HB EGF, and amphiregulin  [ **AREG** ]  were detected in both carcinomas and normal mucosa, indicating that autocrine growth stimulation of colorectal carcinomas is mediated by coexpression of EGF receptor ligands and upregulation of EGF receptors.  Ref: 12790326 Recent Results Cancer Res, 2003
- In this study, we examined the effects of EGF, TGFalpha, amphiregulin  [ **AREG** ]  and heregulin alpha HRGalpha on proliferation, wound closure, and the expression of their receptors c erbB1  [ **EGFR** ]  c erbB4 in primary cultures of human urothelial cells in vitro.  Ref: 12202942 Urol Res, 2002
- 5 OH 2D3 did not affect the levels of the proteoglycan dependent  **EGFR**  ligands amphiregulin  [ **AREG** ]  and heparin binding EGF nor the synthesis of proteoglycans, as assessed by 35S labeling and ion exchange chromatography.  Ref: 9927298 Endocrinology, 1999
- We conclude that amphiregulin  [ **AREG** ]  acts as an autocrine growth factor in HCA 7 and Caco 2 cells, and  **EGFR**  ligand induced proliferation is influenced by cellular polarity.  Ref: 10362109 Br J Cancer, 1999
- Reverse transcription polymerase chain reaction was performed on each sample with primers for rat epidermal growth factor, epidermal growth factor receptor  [ **EGFR** ]  ErbB  [ **EGFR** ] , heparin binding epidermal like growth factor, hepatocyte growth factor, hepatocyte growth factor receptor, keratinocyte growth factor, betacellulin, amphiregulin  [ **AREG** ] , and neuregulin alpha.  Ref: 12148846 Laryngoscope, 2002
- Up regulation of transmembrane tyrosine kinase receptors c met, IGFR1, IGFR2 and  **EGFR**  family related ligands such as HB EGF, TGFa, amphiregulin  [ **AREG** ]  and neuregulin as well as c met ligand HGF point to a possible role of this system in metastasis.  Ref: 12174879 Anticancer Res< MedlineTA>Anticancer Res, 2002
- **EGFR**  mRNA was detected in all tumours, transforming growth factor alpha TGFalpha mRNA levels were similar to those in normal bladder urothelia or were decreased and mRNA levels for amphiregulin  [ **AREG** ] , heparin binding epidermal growth factor like factor HB EGF and betacellulin were significantly higher than those in normal urothelia.  Ref: 11133810 Carcinogenesis, 2000
